# Efficacy and Safety of Ashwagandha Root Extract on Cognitive Functions in Healthy, Stressed Adults: A Randomized, Double-Blind, Placebo-Controlled Study

**DOI:** 10.1155/2021/8254344

**Published:** 2021-11-30

**Authors:** Kumarpillai Gopukumar, Shefali Thanawala, Venkateswarlu Somepalli, T. S. Sathyanaryana Rao, Vijaya Bhaskar Thamatam, Sanjaya Chauhan

**Affiliations:** ^1^Bengaluru Neuro Centre, Bengaluru, Karnataka 560003, India; ^2^Inventia Healthcare Ltd., Mumbai, Maharashtra 400069, India; ^3^Laila Nutraceuticals, Vijayawada, Andhra Pradesh 520007, India; ^4^Sumana Vinayaka Nursing Home, Mandya, Karnataka 571401, India; ^5^In Vitro Research Solutions (iVRS) Pvt Ltd., Bengaluru, Karnataka 560092, India

## Abstract

**Background:**

The global prevalence of stress is increasing. Stress adversely affects cognitive ability, sleep quality, and overall psychological well-being. Ashwagandha (*Withania somnifera* (L.) Dunal), an essential medicine in Ayurveda, is reportedly beneficial in reducing stress and improving memory. This double-blind, randomized, placebo-controlled clinical study evaluated the effect of Ashwagandha root extract sustained-release capsule 300 mg (Prolanza™; hereafter Ashwagandha SR) on cognitive functions, stress levels, sleep quality, overall well-being, and safety in stressed subjects.

**Methods:**

Subjects (130 healthy cognitively sound adults [20–55 years, body mass index:18–29 kg/m^2^]) having a Perceived Stress Scale (PSS) score of 14–24 were randomized to receive either Ashwagandha SR or placebo. Subjects took one capsule of Ashwagandha SR or placebo daily for 90 consecutive days. This study was registered on Clinical Trials Registry-India (CTRI) on 13/11/2019 [number: CTRI/2019/11/021990]. The primary endpoint was the change in cognitive function as measured by CANTAB from baseline to the end of the study period (90 ± 7 days). The secondary outcomes included the change in PSS-10 score, serum cortisol level (9–11 am), the OHQ score, the PSQI, and serum BDNF levels.

**Results:**

Only 125 completed the study and were evaluated. The Cambridge Neuropsychological Test Automated Battery (CANTAB) reported significantly improved recall memory, and the total error rate in recalling patterns significantly decreased at visit 4 in the Ashwagandha SR group vs. the placebo group (first attempt memory score:12.9 ± 6.7 vs. 10.1 ± 6.3; total errors:17.5 ± 23.3 vs. 27.7 ± 23.6). At visit 4, lower PSS-10 score (13.0 ± 5.0 vs. 18.7 ± 4.6; *p* < .0001), serum cortisol levels (*p*=0.0443), and Pittsburgh Sleep Quality Index (PSQI) score (*p* < .0001) but higher Oxford Happiness Questionnaire (OHQ) scores (*p* < .0001) were seen in Ashwagandha SR vs. the placebo group, suggesting significantly lower stress levels and significantly better psychological well-being and sleep quality in the former. No adverse events were reported.

**Conclusions:**

This is the first clinical study assessing Ashwagandha SR for its safety and efficacy. Treatment with one Ashwagandha SR capsule once daily for 90 days improved memory and focus, psychological well-being, and sleep quality, reduced stress levels, and was safe and well-tolerated.

## 1. Introduction

Stress is omnipresent, and its prevalence is increasing globally [1]. Among adults, experiences of the past, occupation, family problems, and environmental factors act as some of the sources of stress. The COVID-19 pandemic has further added to the prevalence and severity of stress [1]. According to a recent systematic review, the prevalence of stress among the general population during the 2020 pandemic was 29.6% (95% confidence limit [CI]: 24.3%–35.4%) [1]. Thus, stress is a major risk factor for the development of both physical and psychiatric conditions. It adversely affects cognitive ability, memory, sleep quality, and overall psychological well-being of individuals besides affecting executive functions, all these together leading to the development of somatic problems [2].

Stress is generally expressed as a psychological or physical response to internal or external stimuli that challenge one's resources for adaptation [3]. The deleterious effects of stress are thought to be channeled through dynamic responsiveness of the hypothalamic-pituitary-adrenal (HPA) axis, resulting in increased cortisol secretion by the adrenal cortex in response to stressors [4]. Cortisol initiates numerous physiological changes to compensate for the additional demands, and prolonged overactivation or suppression of the HPA axis may harm both physical and mental health [4]. The HPA axis also influences sleep: HPA activation leads to disturbed and lighter sleep, and inadequate sleep increases the HPA axis's basal activity [4]. Indeed, factors such as subtle HPA axis abnormalities and higher trait anxiety are associated with lower sleep quality and poorer cognitive functioning among healthy adults [5].

There is currently a global trend of widespread use of complementary and alternative medicine (CAM) in developing and developed countries for the treatment of various ailments such as osteoarthritis, Alzheimer's disease, uremic pruritus, and stress and anxiety [6–9]. Therefore, it is conceivable that a high proportion of individuals may opt to use CAM to manage stress and its effects. A systematic literature review of 49 surveys conducted across 15 countries reported that the 12-month estimated prevalence of any CAM use was approximately 50%, 40%, and 25% in Australia, the United States, and the United Kingdom, respectively [10]. The prevalence of CAM use was generally higher in adults and the elderly. A nationally representative survey conducted by the National Center for Health Statistics in the USA demonstrated a 40% prevalence of CAM use; among these, 18% of the population used natural adaptogens for managing their stress and anxiety [11].

Ayurveda, a traditional system of medicine practiced in India for thousands of years, recommends the use of herbal therapies to aid memory and cognition [12]. Recent evidence shows that plant-derived products may enhance memory and cognition [13]. The root extract of Ashwagandha (*Withania somnifera* (L.) Dunal) is an essential therapeutic agent used in Ayurveda [14] as a “Rasayana” or an “adaptogen” [15]. It is also known as Indian ginseng or winter cherry. Besides improving memory and cognition, it also has antistress, antioxidant, immunomodulatory, rejuvenating, anti-inflammatory, antiarthritic, and antitumor effects [15].

Current evidence supporting the efficacy and safety of Ashwagandha root extract on cognitive functions or stress is from small studies. A recent randomized, double-blind, placebo-controlled trial of 50 adults with mild cognitive impairment showed a significant improvement in immediate and general memory, executive function, attention, and the speed of processing information over an 8-week follow-up period [16]. A prospective, double-blind, randomized, placebo-controlled trial conducted with 61 adults over 60 days showed significant reductions in multiple efficacy parameters such as the Perceived Stress Scale (PSS), General Health Questionnaire-28, and Depression Anxiety Stress scale scores, and cortisol levels in the Ashwagandha group compared to the placebo group [17]. However, the main limitation of these trials was their small sample sizes, thus necessitating a need for larger trials assessing the benefits of Ashwagandha. Also, these previous trials used a twice-daily administration of a high dose (300 mg) of Ashwagandha extract. High dosing frequency can affect long-term compliance, which can, in turn, affect the efficacy of the product.

Our prospective, randomized, double-blind, placebo-controlled clinical study was designed to evaluate the efficacy of the sustained-release (SR) formulation of the Ashwagandha root extract (Ashwagandha SR capsule 300 mg) on cognitive functions, stress level, sleep, and quality of life in subjects experiencing stress. We also assessed the safety and tolerability of Ashwagandha SR capsules.

## 2. Materials and Methods

### 2.1. Study Participants

The study was conducted from December 2019 to May 2020 at two centers in India: Bengaluru Neuro Center, Bengaluru; Sumana Vinayaka Nursing Home, Mandya. Healthy, cognitively sound adult male or female subjects (age: 20–55 years; body mass index [BMI]: 18–29 kg/m^2^) who perceived themselves to be under stress and had a score of 14–24 on the 10-item PSS were included. The inclusion criteria were as follows: men and women aged 20–55 years, body mass index, 18–29 kg/m^2^; healthy as determined by medical history, physical examination, and clinical judgment of the investigator; under perceived stress with a score of 14–24 on the PSS; cognitively sound, as confirmed by the Montreal Cognitive Assessment test; willing and able to provide written informed consent; and agreed to attend regular follow-ups.

Following were the exclusion criteria: women who were pregnant or lactating or those planning to become pregnant and/or unwilling to follow standard contraception methods; postmenopausal women; subjects with an education level below the eighth grade and were unable to read or understand English; subjects with any severe comorbid medical conditions or any clinically significant systemic disease with significant medication use; subjects with a history of drug dependence, high alcohol intake (>2 standard drinks per day), or reported use of recreational drugs (such as cocaine, methamphetamine, and marijuana) or having nicotine or caffeine dependence; subjects on multivitamins/herbal supplements/any other wellness product; subjects who had participated in a clinical study within the last 30 days before enrolling in this study; or subjects with hypersensitivity to any ingredients of the study products.

The study was conducted in accordance with Good Clinical Practice, the Declaration of Helsinki, Schedule Y (amended version 2005), and the Ethical Guidelines for Biomedical Research on Human Subjects, issued by the Indian Council of Medical Research and New Drugs and Clinical Trials Rules 2019. This study was registered on Clinical Trials Registry-India (CTRI) on 13/11/2019 [Registration No.: CTRI/2019/11/021990]. An independent, registered ethics committee (Lifeline Ethics Committee, Bangalore 560079, India) reviewed and approved the protocol before the commencement of the study (ECR/76/Indt/KA/2013/RR-16). Participants provided their written informed consent before enrolment into the study.

### 2.2. Test Product Details

The investigational product (Prolanza™ [Inventia Healthcare Ltd. and Laila Nutraceuticals]) is a sustained-release (SR) capsule containing *Withania somnifera* root aqueous alcohol, n-butanol extract, and permitted excipients. It is formulated to provide sustained release of the actives. Each 300 mg capsule contains total withanolides NLT 15 mg by HPLC. Pharmacokinetic data like relative absorption, relative bioavailability, and elimination half-life are evaluated and submitted for publication elsewhere.

### 2.3. Overview of the Study Design

**Figure 1:**
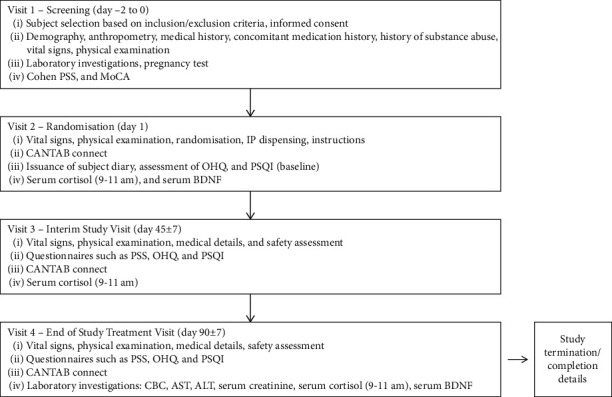
Overview of study design. ALT, alanine aminotransferase; AST, aspartate aminotransferase; BDNF, brain-derived neurotrophic factor; CANTAB, Cambridge Neuropsychological Test Automated Battery; CBC, complete blood count; IP, investigational product; OHQ, Oxford Happiness Questionnaire; PSQI, Pittsburgh Sleep Quality Index; PSS, Perceived Stress Scale; MoCA, Montreal Cognitive Assessment.

TA 90-day prospective clinical study was conducted at two study centers using a double-blind, randomized, parallel-group, two-arm, placebo-controlled design. The participants completed four study visits (Figure 1). The first visit (day −2 to 0) was for screening purposes, wherein subjects meeting inclusion criteria were identified, and informed consent was obtained. We collected data on subject history and baseline PSS score and completed the preliminary laboratory tests in this visit. During the second visit (day 1), we randomized subjects to the Ashwagandha SR or the placebo group in a double-blinded manner. An independent statistician generated the randomization sequence. Product labels were replaced with the label containing a computer-generated blinded sequence created by a statistician for the purpose of blinding the subjects and investigators. After completion of the analysis (completion of the data lock process), the analyst used the same list to decode the groups.

The investigational product (Ashwagandha SR capsules or the look-alike placebo) was provided to the subjects, and they were instructed to take one capsule daily after breakfast for 90 consecutive days. We also instructed them to maintain the subject diary by entering the date, checking the box and entering the time they took the capsule, and making a note if they miss a capsule (for days 1–45). During this visit, we collected baseline data for Cambridge Neuropsychological Test Automated Battery (CANTAB), Oxford Happiness Questionnaire (OHQ) [18], and Pittsburgh Sleep Quality Index (PSQI) [19] scores and assessed serum cortisol (9–11 am) and brain-derived neurotrophic factor (BDNF) levels. The third visit was an interim visit (day 45) when all these data were collected again, the subject diary was checked for compliance, and the investigational products were provided for the next 45 days. Additionally, one more subject diary was given to the subjects to record compliance. Visit 4 (day 90) was the final visit when the final assessments of efficacy and safety endpoints were completed, and the subject diary was checked for compliance. Figure 1 provides an overview of the study design and details of procedures at each visit.

### 2.4. Study Endpoints

#### 2.4.1. Efficacy Endpoints

The primary endpoint was the change in cognitive function as measured by CANTAB from baseline to the end of the study period.

The secondary outcomes included the change from baseline to the end of the study period in PSS-10 score, serum cortisol level (9–11 am), the OHQ score, the PSQI, and serum BDNF levels.

#### 2.4.2. Safety Endpoints

Safety was assessed as the proportion of subjects who discontinued the study treatment due to adverse events (AEs; their frequency and severity), such as changes in vital parameters and laboratory investigations (complete blood count [CBC], alanine aminotransferase [ALT], aspartate aminotransferase [AST], and serum creatinine levels).

### 2.5. Study Assessments

#### 2.5.1. Cambridge Neuropsychological Test Automated Battery (CANTAB)

The CANTAB is a validated scale originally designed to assess cognitive function in the elderly and populations with dementia [20]. However, it has been utilized for studying various populations [21], including normal functioning adults [20]. The CANTAB includes tests of working memory; learning and executive function; visual, verbal, and episodic memory; attention, information processing, and reaction time; social and emotion recognition and decision making; and response control. We used the CANTAB general cognition battery comprising the following five tests, often used in clinical research to identify subtle cognitive changes: Motor Screening Test (MOT), Paired Associates Learning (PAL), Reaction time (RT), Rapid Visual Information Processing (RVP), and Spatial Working Memory (SWM).

#### 2.5.2. Perceived Stress Scale-10 (PSS-10)

The PSS-10 is a validated self-reported questionnaire designed to measure “the degree to which individuals appraise situations in their lives as stressful.” The PSS items evaluate the degree to which individuals believe their life has been unpredictable, uncontrollable, and overloaded during the previous month [22]. Scores of around 13 are considered average, and those of ≥20 reflect high stress.

#### 2.5.3. Serum Cortisol Level (9–11 am)

Serum cortisol is a glucocorticoid hormone, the marker of the body's response to stress. It is secreted in response to biochemical stress and contributes to the suppression of the HPA axis, affecting health and cognition [23]. Owing to the known diurnal variations, serum cortisol levels (in *μ*g/dL) were measured at a fixed time slot between 9 and 11 am [24].

#### 2.5.4. Oxford Happiness Questionnaire (OHQ)

The OHQ is a compact scale used for measuring psychological well-being [18]. It is presented as a single statement that can be endorsed on a uniform six-point Likert scale. Based on the score, it categorizes happiness levels as follows: 1-2: not happy, 2-3: somewhat unhappy, 3-4: not particularly happy or unhappy, 4: somewhat happy or moderately happy, 4-5: rather happy; pretty happy, 5-6: very happy, and 6: too happy.

#### 2.5.5. The Pittsburgh Sleep Quality Index (PSQI)

The PSQI is an effective instrument for measuring the quality and patterns of sleep 1 month before study initiation. It differentiates “poor” from “good” sleep by measuring seven domains: subjective sleep quality, sleep latency, sleep duration, habitual sleep efficiency, sleep disturbances, use of sleep medication, and daytime dysfunction over the past month [19]. Subjects score each domain on a 0–3 Likert scale, where 0 reflects no difficulty and 3 reflects the severe difficulty in that domain. The global score (range 0 to 21) is the sum of component domain scores. Higher scores indicate worse sleep quality. We assessed PSQI in both groups at visit 2 (baseline) and visit 4.

#### 2.5.6. Serum Brain-Derived Neurotropic Factor (BDNF)

The BDNF is a nerve growth agent that supports, grows, and differentiates the existing neurons of the central and peripheral nervous systems. Stress likely affects the expression of BDNF in different parts of the brain in a manner that is highly sensitive to the form, duration, and timing of stress and the sex of the subject [25]. We assessed BDNF in both groups at visit 2 (baseline) and visit 4 by using the Sandwich enzyme-linked immunosorbent assay (LSBio, Seattle, WA; Human BDNF Sandwich ELISA [LS-F678]) [26].

### 2.6. Statistical Analysis

To see improvement in one of the cognition tests of CANTAB, an effect size of Cohen's *d* = 0.5 (i.e., Cohen's *f* = 0.25, as *d* = 2f) at *α* = 0.05 was utilized. To detect this effect size at a power of 80% and a level of significance of 0.05, the sample size was estimated to be 128 subjects (64 subjects in each group). The moderate effect size was chosen based on similar nutritional intervention studies that have detected significant improvements in cognitive outcomes that may indicate restoration of cognitive function [27, 28].

Statistical analysis was performed of the data obtained from the subjects who completed the study, with compliance of >80% according to the protocol (modified intension-to-treat). The summary statistics are presented as mean ± standard deviation. An ANOVA was used to assess the within-treatment differences for efficacy variables from baseline to the end of treatment. For between-treatment analyses, an ANCOVA was used to assess the differences in efficacy variables at the end of the treatment using baseline values as covariates. *p* values are reported for all the efficacy parameters. A *p* < 0.05 was considered as the threshold for statistical significance.

## 3. Results

### 3.1. Study Flow

Of the 147 subjects screened, 130 subjects (age, 34.3 ± 9.0 years; BMI, 24.59 ± 3.49 kg/m^2^) who met the inclusion criteria were randomized; 65 (34 men and 31 women) were in the Ashwagandha SR group and 65 (36 men and 29 women) in the placebo group. Five subjects withdrew their consent voluntarily during the COVID-19 lockdown period.

**Figure 2:**
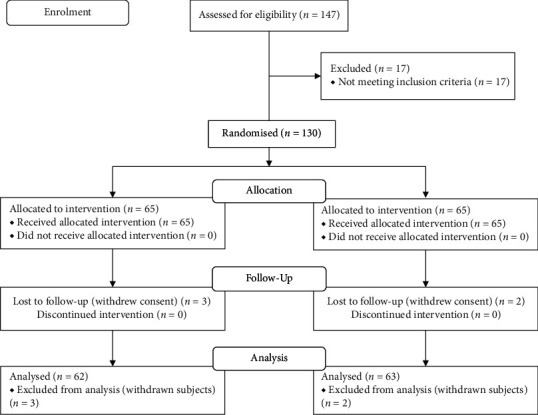
Consolidated Standards of Reporting Trials (CONSORT) flow diagram.

A total of 125 subjects completed the study and comprised the evaluation population; all subjects were compliant with the protocol.  Figure 2 provides a Consolidated Standards of Reporting Trials (CONSORT) flow diagram of the study.

### 3.2. Efficacy Analysis

#### 3.2.1. CANTAB Score

**Figure 3:**
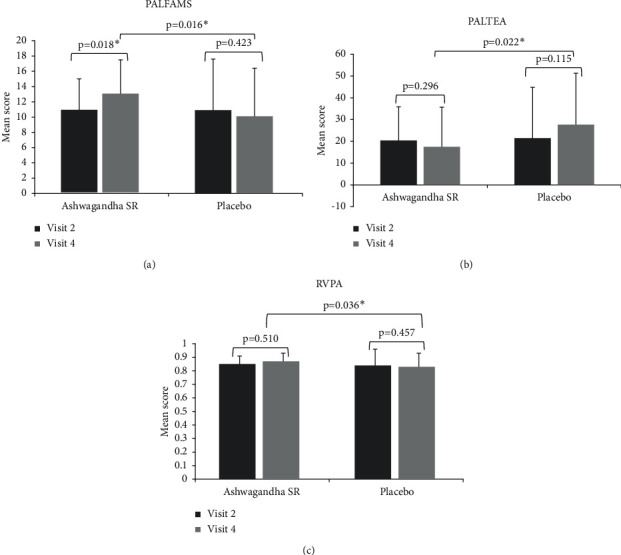
Within-group and between-group differences for CANTAB scores of PALFAMS (a), PALTEA (b), and RVPA (c) from baseline to the last visit. CANTAB, Cambridge Neuropsychological Test Automated Battery; PAL, Paired Associates Learning; PALFAMS, PAL First Attempt Memory Score; PALTEA, PAL Total Errors (Adjusted); RVPA, Rapid Visual Information Processing A Prime.

The within- and between-group differences for CANTAB scores are shown in Table 1 (supplementary file). CANTAB showed that recalling memory significantly improved, and the total error rate in recalling patterns significantly decreased at visit 4 in the Ashwagandha SR group compared with the placebo group. For Ashwagandha SR group, PAL First Attempt Memory Score (PALFAMS; mean ± SD) significantly increased from 10.8 ± 4.2 at visit 2 to 12.9 ± 6.7 at visit 4 (*p* < 0.05; Figure 3(a)), while adjusted PAL Total Errors (Adjusted) (PALTEA) decreased from 20.4 ± 15.4 at visit 2 to 17.5 ± 23.3 at visit 4 (*p* > 0.05; Figure 3(b)). In contrast, in the placebo group, the results showed a numerical nonsignificant decrease in recalling memory (PALFAMS, 10.9 ± 4.6 vs. 10.1 ± 6.3) and an increase in error rate (PALTEA, 21.5 ± 18.2 vs. 27.7 ± 23.6) from visit 2 to visit 4. Thus, at visit 4, the Ashwagandha SR group scored significantly higher for recalling memory (PALFAMS: 12.9 ± 6.7 vs. 10.1 ± 6.3; *p* < 0.05) and lower for total errors (PALTEA: 17.5 ± 23.3 vs. 27.7 ± 23.6; *p* < 0.05) than the placebo group after adjusting for baseline values (Figures 3(a) and 3(b)). Different components of reaction time showed significant increases from visit 2 to visit 4 in both the groups, but the differences at visit 4 after adjusting for baseline scores were not significant, as represented in Table 1 (supplementary file). Also, the differences in the Ashwagandha SR group, compared with those in the placebo group, at visit 4 were not significant for MOTML, RVP (except RVPA), and SWM parameters as represented in Table 1 (supplementary file). For RVPA, the Ashwagandha SR group scored significantly higher (0.87 ± 0.12) than the placebo group (0.83 ± 0.10 at visit 4 (*p* < 0.05, after adjusting for baseline scores; Figure 3(c)).

#### 3.2.2. PSS-10 Score

The PSS-10 score for the Ashwagandha SR group significantly reduced from 19.5 ± 3.2 to 13.0 ± 5.0 from visit 1 to visit 4, respectively (*p* < .0001; Table 1). The corresponding scores for the placebo group were 19.4 ± 3.1 and 18.7 ± 4.6, but the difference was not significant (*p*=0.4955). At visit 4, a statistically significant difference in the PSS-10 score was observed in the Ashwagandha SR compared with the placebo group (*p* < .0001). A Cohen *d* of 1.55 for the PSS score in the Ashwagandha SR group, compared with 0.18 in the placebo group, also corroborates the effect of Ashwagandha SR. Cohen *d* of 1.4 (1.55–0.18) translates to a probability of 83.4% superiority of the Ashwagandha SR capsule compared to the placebo.

#### 3.2.3. Serum Cortisol Level (9–11 Am)

The serum cortisol level (*μ*g/dL) for the Ashwagandha SR group reduced significantly from 9.04 ± 3.77 *μ*g/dL to 6.34 ± 2.31 *μ*g/dL from visit 2 to visit 4, respectively (*p* < .0001; Table 1). Similarly, the serum cortisol level decreased significantly in the placebo group as well (from 9.01 ± 3.69 *μ*g/dL at visit 2 to 7.38 ± 3.31 *μ*g/dL at visit 4; *p* = 0.0078). At visit 4, the serum cortisol level in the Ashwagandha SR group was significantly lower than that in the placebo group (*p* = 0.0443).

#### 3.2.4. OHQ Score

The OHQ score in the Ashwagandha SR group increased significantly from 3.93 ± 0.60 at visit 2 to 4.39 ± 0.78 at visit 4 (*p*=.0003; Table 1). In contrast, the placebo group showed a significant reduction in the OHQ score, from 3.91 ± 0.77 to 3.44 ± 0.69, during the same period. At visit 4, the OHQ score was significantly higher in the Ashwagandha SR group compared with the placebo group (*p* < .0001).

#### 3.2.5. PSQI Score

The mean PSQI score in the Ashwagandha SR group decreased significantly from 4.6 ± 2.7 at visit 2 to 2.5 ± 1.6 at visit 4 (*p* < .0001; Table 1). The mean PSQI score for the placebo group was 4.6 ± 2.2 and 4.0 ± 2.0 at visit 2 (baseline) and visit 4, respectively; however, the difference was not statistically significant (*p*=0.08). Also, at visit 4, the PSQI in the Ashwagandha SR group was significantly lower than in the placebo group (*p* < .0001).

#### 3.2.6. Serum BDNF Levels

Serum BDNF levels were not significantly different in the Ashwagandha SR compared with the placebo group at visit 4: the levels were 503.43 ± 216.74 pg/mL and 490.68 ± 207.75 pg/mL, respectively (*p*=0.7407).

### 3.3. Safety Analysis

Changes in safety parameters (CBC, AST, ALT, and serum creatinine levels) were assessed from baseline (visit 1) to visit 4 (day 90). Both groups showed no abnormal changes, and there were no observed AEs during the entire study period. Five subjects withdrew voluntarily from the study after visit 3. No protocol violations were observed, and protocol deviations were minor.

## 4. Discussion

In this randomized placebo-controlled clinical study, we assessed the effect of Ashwagandha SR capsule (300 mg) on cognitive functions, stress level, sleep quality, and overall well-being of subjects reporting stress, as well as assessing the safety of the test product. Research has shown that stress affects cognitive abilities and can result in various disorders such as coronary heart disease, hypertension, diabetes, infectious diseases, and affective and anxiety disorders [2]. The current study demonstrated the benefits of Ashwagandha SR in improving cognition while reducing stress levels and improving sleep quality. This dual efficacy on cognitive abilities and sleep quality observed with Ashwagandha SR is beneficial, especially when considering the adverse effects of traditional anxiolytics (e.g., benzodiazepines) that cause sedation and impair cognitive abilities [29].

We demonstrated that subjects receiving the Ashwagandha SR capsule performed better in the three domains of cognitive skills, namely, visual memory, new learning, and working and sustained attention. CANTAB scores showed that recalling memory (PALFAMS) significantly improved, and the total error rate (PALTEA) in recalling patterns significantly decreased from baseline to visit 4 in the Ashwagandha SR group but not in the placebo group. Also, the Ashwagandha SR group had a significantly higher RVPA score than the placebo group at visit 4, indicating better sensitivity for detecting the target sequences in the former group.

Further, subjects receiving the Ashwagandha SR capsule had an 83.4% greater probability of achieving control over their stress, as measured by the PSS-10 scores. The significantly lower serum cortisol level in the Ashwagandha SR group, compared with that in the placebo group, at visit 4 substantiates these results of PSS-10 scores. Additionally, PSQI evaluation showed that both the quality and sleep pattern improved significantly in the Ashwagandha SR group but not in the placebo group. Interestingly, the Ashwagandha SR group also demonstrated higher OHQ scores, suggesting improvement in psychological well-being. Notably, the OHQ scores decreased in the placebo group, suggesting deterioration of psychological well-being in these subjects during the same period.

These benefits were observed during an interesting period. Specifically, our study was conducted from December 02, 2019, to May 26, 2020. Thus, a substantial proportion (72%) of the study subjects underwent the final assessment (at 90±7days) after the World Health Organization declared COVID-19 as a pandemic on March 11, 2020 [30], or after the COVID-19 lockdown period (from the first to the fourth phase, between March 24, 2020, and May 31, 2020) was imposed by the Government of India [31]. Overall, research shows that stress and anxiety levels were high during the lockdown period, and sleep and psychological well-being had deteriorated. An online survey conducted in April 2020 assessed the psychological impact of lockdown due to the COVID-19 pandemic on the general public (*N* = 1685). The proportion of stress, poor well-being, anxiety, and depression was high: 74.1% of respondents reported moderate levels of stress, 71.7% reported poor well-being, 38.2% had anxiety, and 10.5% had depression [32]. Another online survey conducted in early May 2020 concluded that the quality and quantity of night sleep deteriorated across groups during the lockdown, and reduction in sleep duration was associated with depressive symptoms [33]. Our study highlights the positive effects of Ashwagandha SR in reducing stress and improving overall psychological well-being and sleep quality during these testing times, further supporting the product's efficacy.

Several studies have evaluated the therapeutic effects of Ashwagandha on stress, anxiety, and cognitive abilities and have shown significant improvements in stress, mood, or cognitive function across populations [16, 17]. However, the sample sizes of these studies were smaller than that of the present study. Furthermore, these studies often used twice-daily doses for Ashwagandha formulations, whereas we used a once-daily dose of an SR capsule. Our formulation has better bioavailability of active constituents of Ashwagandha for an extended period, as shown in our comparative pharmacokinetic study wherein the relative bioavailability for the Ashwagandha SR formulation was 12, 44, and 11 times higher for total withanolides, withanolide A, and 12-deoxywithastramonolide, respectively, compared with the reference formulation (unpublished data, submitted for publication). Thus, our formulation has the potential to improve cognitive levels while maintaining safety. As simpler and less frequent dosing regimens result in better compliance across therapeutic classes [34], a single daily dose of Ashwagandha SR capsule is also expected to increase compliance.

The safety and tolerability of the Ashwagandha SR capsules were also demonstrated in our study. No adverse events, minor protocol deviations, no violations, and >80% compliance to treatment confirm the safety of Ashwagandha SR capsules. The minor protocol deviations include few subjects not falling in recommended BMI range, deviations in the CANTAB assessment schedule due to COVID-restrictions.

Overall, this study, with its sample size based on adequate power, expands the current evidence that has been derived mostly from smaller studies. A major strength of the study is that it evaluated a sustained-release formulation of Ashwagandha root extract, which is sparingly reported in the literature. Furthermore, our study demonstrated the efficacy of the Ashwagandha SR capsule by using the objective outcome of improvement in cognition. Further studies with a larger pool of subjects and for a longer duration are required to further substantiate the results of this study. Also, further research evaluating the efficacy and safety of Ashwagandha SR in different populations is needed to ensure the generalizability of our findings.

## 5. Conclusions

In this first reported clinical study assessing the “sustained-release formulation” of Ashwagandha root extract, we demonstrated that consumption of Ashwagandha SR capsules for 90 days resulted in improved memory and focus, psychological well-being, and sleep quality and reduced stress levels. We also showed that Ashwagandha SR capsules were safe when administered over the period of 90 days. Thus, these capsules can be used in a single daily dose as an effective adaptogen to improve cognitive abilities and reduce stress in otherwise healthy adults.

## Figures and Tables

**Table 1:** Within-group and between-group differences for additional efficacy parameters from baseline to the last visit.

Index	Baseline visit^§^	Visit 4	*p* value; change from baseline to visit 4	Baseline	Visit 4	*p* value; change from baseline to visit 4	*p* value; the difference in Ashwagandha SR vs. placebo groups at visit 4
	Ashwagandha SR			Placebo			
PSS-10	19.5 ± 3.2	13.0 ± 5.0	<.0001	19.4 ± 3.1	18.7 ± 4.6	*p*=0.4955	<.0001
Serum cortisol, *μ*g/dL	9.04 ± 3.77	6.34 ± 2.31	<.0001	9.01 ± 3.69	7.38 ± 3.31	*p*=.0078	*p*=0.0443
OHQ	3.93 ± 0.60	4.39 ± 0.78	*p*=.0003	3.91 ± 0.77	3.44 ± 0.69	*p*=.0001	<.0001
PSQI	4.6 ± 2.7	2.5 ± 1.6	<.0001	4.6 ± 2.2	4.0 ± 2.0	*p*=0.0794	<.0001

^§^Baseline for PSS-10 assessment was visit 1 and that for serum cortisol, OHQ, and PSQI was visit 2.

## Data Availability

The data sets used or analyzed during the current study are available from the corresponding author on reasonable request.

## Supplementary Materials

Supplementary Materials The within- and between-group differences for CANTAB scores from baseline to last visit are shown in the supplementary file.
