# Supplementary material for: Efficacy and Safety of Ashwagandha Root Extract on Cognitive Functions in Healthy, Stressed Adults: A Randomized, Double-Blind, Placebo-Controlled Study
Source: Evid Based Complement Alternat Med. 2021 Nov 30;2021:8254344. doi: 10.1155/2021/8254344 (PMC8632422; doi:10.1155/2021/8254344)
Supplement: Supplementary Materials — The within- and between-group differences for CANTAB scores from baseline to last visit are shown in the supplementary file. [file 8254344.f1.zip › 8254344.f1/Supplementary file.docx]

**Additional Table 1: Within-group and between-group differences for CANTAB scores from baseline to the last visit**

| **Index** | **Visit 2**  **(n=65)** | **Visit 4**  **(n=62)** | **p value; change between visit 2 and visit 4** | **Visit 2**  **(n=65)** | **Visit 4**  **(n=63)** | **p value; change between visit 2 and visit 4** | **p value; difference in the Ashwagandha vs placebo groups at visit 4** |
| --- | --- | --- | --- | --- | --- | --- | --- |
|  | **Ashwagandha SR group** | | | **Placebo group** | | |  |
| **MOTML** | 984.30±443.16 | 919.20±312.73 | 0.3336 | 962.27±312.74 | 930.43±386.61 | 0.5986 | 0.8878 |
| **PAL** |  |  |  |  |  |  |  |
| PALFAMS | 10.8±4.2 | 12.9±6.7 | 0.0179 | 10.9±4.6^§^ | 10.1±6.3 | 0.4233 | 0.0163 |
| PALTEA | 20.4±15.4 | 17.5±23.3 | 0.2962 | 21.5±18.2^§^ | 27.7±23.6 | 0.1149 | 0.0215 |
| **RT** |  |  |  |  |  |  |  |
| RTIFMDMT | 243.78±59.87 | 251.68±50.00 | 0.1687 | 245.05±62.50 | 269.25±65.86 | 0.0377 | 0.0843 |
| RTIFMDRT | 385.28±46.64 | 439.48±53.59 | <0.0001 | 386.52±59.01 | 436.79±49.89 | <0.0001 | 0.7778 |
| RTISMDMT | 206.60±62.42 | 234.47±53.25 | 0.0006 | 214.29±66.66 | 253.18±58.50 | 0.0002 | 0.0645 |
| RTIFMMT | 246.97±59.48 | 254.47±47.13 | 0.1811 | 249.28±63.43 | 274.32±64.33 | 0.0263 | 0.0440 |
| RTISMDRT | 343.00±48.54 | 415.62±68.11 | <0.0001 | 349.82±69.56 | 426.29±76.79 | p<0.0001 | 0.3946 |
| **RVP** |  |  |  |  |  |  |  |
| RVPA | 0.85369±0.06217^£^ | 0.86751±0.12348 | 0.5097 | 0.84282±0.05626^£^ | 0.82898±0.09800 | 0.4571 | 0.0356 |
| RVPMDL | 476.98±136.25^£^ | 514.88±247.88 | 0.2414 | 481.99±128.62 ^£^ | 527.29±224.04 | 0.2430 | 0.7089 |
| RVPPFA | 0.05195±0.09335^£^ | 0.05453±0.07468 | 0.7096 | 0.08387±0.11593^£^ | 0.07681±0.08745 | 0.6999 | 0.1088 |
| **SWM** |  |  |  |  |  |  |  |
| SWMBE4 | 1.5±1.6 | 1.6±1.4 | 0.7652 | 1.4±1.4 | 1.7±1.7 | 0.2679 | 0.7296 |
| SWMBE6 | 5.4±3.1 | 6.4±4.2 | 0.2533 | 5.0±3.3 | 5.5±3.7 | 0.3930 | 0.1630 |
| SWMBE8 | 11.2±5.3 | 12.8±7.0 | 0.1563 | 11.5±6.3 | 12.2±7.4 | 0.6815 | 0.6555 |
| SWMS | 9.0±1.8 | 9.3±1.8 | 0.6729 | 8.8±2.4 | 9.2±1.8 | 0.2078 | 0.8628 |
| SWMBE | 18.1±7.8 | 21.8±7.9^¥,$^ | 0.0041 | 17.9±9.2 | 19.7±8.8^£,$^ | 0.1403 | 0.1527 |

^§^n=63, ^£^n=64, ^¥^n=65, ^$^Visit 3

PAL, Paired Associates Learning; PALFAMS, PAL First Attempt Memory Score; PALTEA, PAL Total Errors (Adjusted); RT, Reaction Time; RTIFMDMT, RTI Median Five-Choice Movement Time; RTIFMDRT, RTI Median Five-Choice Reaction Time; RTIFMMT, RTI Mean Five-Choice Movement Time; RTISMDMT, RTI Simple Median Movement Time; RVP, Rapid Visual Information Processing; RVPA, RVP A Prime; RVPMDL, RVP Median Response Latency; RVPPFA, RVP Probability of False Alarm; SWM, Spatial Working Memory; SWMBE, SWM Between Errors; SWMBEx, SWM Between Errors × boxes
